# Identification of Changing Lower Limb Neuromuscular Activation in Parkinson's Disease during Treadmill Gait with and without Levodopa Using a Nonlinear Analysis Index

**DOI:** 10.1155/2015/497825

**Published:** 2015-01-19

**Authors:** Amir Pourmoghaddam, Marius Dettmer, Daniel P. O'Connor, William H. Paloski, Charles S. Layne

**Affiliations:** ^1^Center for Neuromotor and Biomechanics Research (CNBR), Health and Human Performance Department (HHP), University of Houston, 3855 Holman Street, Room 104 Garrison, Houston, TX 77204-6015, USA; ^2^Memorial Bone & Joint Research Foundation, 1140 Business Center Drive, Suite 101, Houston, TX 77043, USA

## Abstract

Analysis of electromyographic (EMG) data is a cornerstone of research related to motor control in Parkinson's disease. Nonlinear EMG analysis tools have shown to be valuable, but analysis is often complex and interpretation of the data may be difficult. A previously introduced algorithm (SYNERGOS) that provides a single index value based on simultaneous multiple muscle activations (MMA) has been shown to be effective in detecting changes in EMG activation due to modifications of walking speeds in healthy adults. In this study, we investigated if SYNERGOS detects MMA changes associated with both different walking speeds and levodopa intake. Nine male Parkinsonian patients walked on a treadmill with increasing speed while on or off medication. We collected EMG data and computed SYNERGOS indices and employed a restricted maximum likelihood linear mixed model to the values. SYNERGOS was sensitive to neuromuscular modifications due to both alterations of gait speed and intake of levodopa. We believe that the current experiment provides evidence for the potential value of SYNERGOS as a nonlinear tool in clinical settings, by providing a single value index of MMA. This could help clinicians to evaluate the efficacy of interventions and treatments in Parkinson's disease in a simple manner.

## 1. Introduction

For many years, physiologists, physicians, and clinicians have employed various techniques to understand how the brain and, in more general terms, the central nervous system (CNS) control human movement. This complex system generates different patterns of neuromuscular activity associated with certain environmental conditions during performance of various motor tasks [1–5]. In addition, it is well documented that neuromuscular activities are altered by different factors of physiological health or aging [6, 7]. A deep understanding of the nervous system is necessary for adequate treatment of impairments of the nervous system resulting from neuromuscular disorders, for example, Parkinson's disease [1, 2].

Surface electromyography (EMG) is a technique used to investigate neuromuscular activity associated with movement. The strategies by which the CNS controls human movement can be revealed by studying the collective activation and complex orchestration of motor units in response to changes in external factors and internally generated movement goals [8]. This approach is used extensively in basic research or in clinical and rehabilitation settings, with the goal to evaluate and monitor the state and responses of the neuromuscular system in both healthy and pathological individuals [9–12].

It has been postulated that one movement control strategy used by the CNS is the activation of multiple muscles acting in concert to achieve a specific movement [4, 5, 13, 14]. To investigate this strategy, it is essential to analyze EMG signals from multiple muscles simultaneously in order to determine how neuromuscular activation characteristics are modified under varying conditions. Traditional signal processing analyses (i.e., EMG analyses) have shown diagnostic responsiveness such as detecting neuromuscular fatigue but may be limited in detection of multiple other physiological characteristics and changes thereof [15]. This has likely prevented investigators from detecting subtle but significant changes in neuromuscular activities during initiation and progression of neuromuscular disorders [16, 17]. For a better understanding of symptoms of disease, earlier detection, or the potentially subtle effects of treatments and interventions, sophisticated nonlinear analysis tools are required.

**Figure 1:**
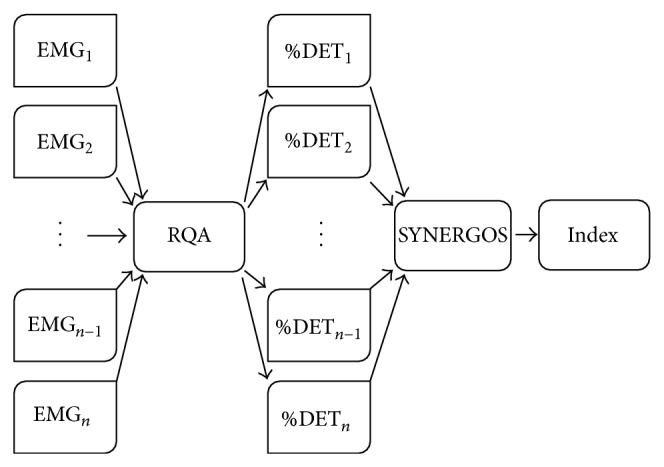
The schematic view of the SYNERGOS algorithm. EMG signals are analyzed using the RQA, and the output for each muscle, %DET, is imported into the SYNERGOS algorithm, which eventually provides a single scalar index representing the state of MMA.

In an earlier publication, we introduced a new analysis technique (SYNERGOS) designed to quantify multiple muscle coactivation (MMA) [18]. SYNERGOS is based on a two-step method for assessing muscular multiple activation quantification. The first step is using Recurrence Quantification Analysis (RQA) to analyze the EMG signals of each recorded muscle separately. The calculated percentage of determinism (%DET) of the EMG signal obtained from each muscle then serves as an input variable for the second step of SYNERGOS in which the inputs are combined by an algorithm that quantifies the level of MMA (Figure 1). A major advantage of the algorithm is the simplicity of the output, represented by a single value. A SYNERGOS index of 100 represents the simultaneous activation of all measured muscles at a theoretical maximum contraction level (100%). Such a situation is extremely unlikely when considering any voluntary contraction and is definitely not possible during a dynamic movement, because absolute rigidity would prevent any dynamic motor task. Lower values indicate less muscular simultaneous activation and a less deterministic nature of activation patterns.

Previously, we demonstrated that SYNERGOS is effective in detecting changes in MMA induced by modifications of task constraints (different walking and running speeds on a treadmill) [18]. As shown in the earlier experiment, changes in the quantity of SYNERGOS reflected changes in the neuromuscular activity characteristics governed by the CNS of healthy individuals, potentially to reduce the number of degrees-of-freedom (DOF) used during motor task performance. Monitoring the SYNERGOS indices provided a quick and simple “snapshot” of the overall neuromuscular coordination and activity generated during a given movement.

As another step to investigate the potential of SYNERGOS as a monitoring tool, it is necessary to test if the novel technique is also sensitive to subtle changes of neuromuscular activation, that is, the level of MMA, for example, in response to pharmaceutical interventions in neuromuscular disease. It is known that Parkinson's disease affects movement and gait patterns, due to the degeneration of motor areas in the brain responsible for generation of coordinated, smooth, and rhythmic motor patterns. However, levodopa intake alters these patterns, as evidenced by biomechanical analysis of gait [19].

Thus, we designed an experiment to investigate the responsiveness of the SYNERGOS algorithm to the changes in neuromuscular activities in individuals with Parkinson's disease after levodopa intake, during treadmill walking with increasing speed. We hypothesized that increasing walking speeds with or without levodopa would be reflected in SYNERGOS as an increase in the index, indicating increased activation of multiple muscles. Further, we hypothesized that levodopa would be associated with a decrease in the SYNERGOS index relative to the no levodopa conditions across the increasing walking speeds, reflecting a more efficient MMA strategy in patients due to lowering the level of simultaneous muscle activities. These findings would demonstrate the potential of the SYNERGOS index to serve as a screening tool for the evaluation of the therapeutic interventions in individuals with neuromuscular disorders or those in rehabilitation settings.

## 2. Methods

Nine men diagnosed with Parkinson's disease participated in this study (Table 1). The subjects were recruited based on three inclusion criteria: (1) a certified neurologist specialized in movement neuromuscular disorders diagnosed the idiopathic Parkinson's disease; (2) the participant was receiving oral anti-Parkinsonian medications; (3) the participant was required to be able to independently walk and be in the early stages of Parkinson's disease, as defined by stages 2 to 3 of the Hoehn and Yahr scale [20]. This study was conducted in compliance with all the regulations of the University of Houston and Baylor College of Medicine and was approved by the Committees for the Protection of Human Subjects (CPHS) at the University of Houston and the Institutional Research Board of Baylor College of Medicine. All participants were adequately provided with instructions to understand the test protocols, risks, and benefits, and written consent was obtained from each participant prior to the start of data collection.

### 2.1. Apparatus

**Figure 2:**
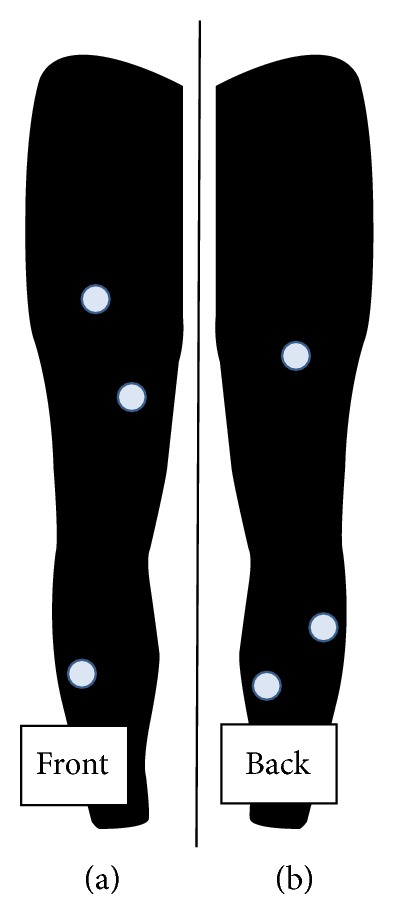
Position of the EMG electrodes on the front of the right leg (a) and back of the right leg (b). Muscle activity was measured at the rectus femoris (RF), vastus medialis (VM), tibialis anterior (TA), biceps femoris (BF), lateral gastrocnemius (GA), and soleus (SO) of the right leg.

All walking experiments were conducted on a motorized treadmill (Biodex Medical Systems Inc., RTM 4000, Shirley, NY). EMG signals were collected using six preamplifier bipolar active electrodes (EMG preamplifier SX230, Biometrics Ltd., Gwent, UK) with a fixed electrode distance of 20 mm placed on specific muscles of the right leg (Figure 2), affixed with double-sided tape and athletic wraps. The electrodes were connected to a DataLINK DLK900 base-unit (Biometrics Ltd., Gwent, UK) of the EMG acquisition system which was connected to a personal computer using a Universal Serial Bus (USB) cable. To achieve acceptable impedance level, the skin over the location of each electrode was shaved and cleaned with alcohol swabs. EMG data were collected at 1000 Hz and passed through an amplifier with the gain set at 1000. The amplification bandwidth was 20–460 Hz (input impedance = 100 MΩ, common mode rejection ratio >96 dB (~110 dB) at 60 Hz). A reference electrode was placed above the right lateral malleolus bone and was secured with elastic wrap and tapes. During the collection session, the electrodes were not removed from the subjects until data collection was completed.

**Figure 3:**
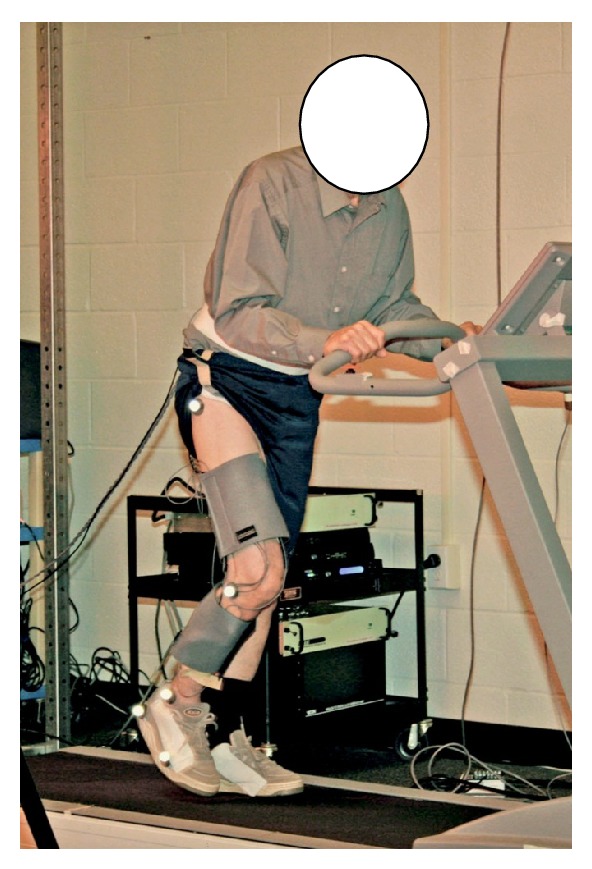
Reflective markers were placed on the hip, knee, ankle, heel, and toe (modified) to calculate the heel contact. In addition, EMG sensors were located on the right rectus femoris, tibialis anterior, lateral gastrocnemius, soleus, vastus medialis, and biceps femoris, and two workout wraps were used to stabilize the EMG sensors during faster gait speed. The subjects were instructed to hold the safety bar installed on the treadmill to avoid excessive anterior-posterior movements.

A 6-camera Vicon motion capture device (Oxford Metrics, Oxford, UK) was used to collect three-dimensional kinematic data from reflective markers that are placed on the right hip, knee, ankle, heel, and toe at 120 Hz to identify gait cycles (gait events) by detecting the position/point-in-time of each heel strike (i.e., right foot heel strike to the next right heel strike). Heel strikes were defined at the point of the maximal anterior position of the heel marker during each gait cycle [19]. Kinematic markers were located on the hip, knee, ankle, heel, and toe. An electronic trigger was used to synchronize EMG and kinematic data and to identify the times at which treadmill speed increased (Figure 3). In this study, we only utilized the kinematics data collected from the heel marker to detect the changes in gait pattern and identify each gait cycle. Identification of gait cycles is required to calculate the SYNERGOS index as the EMG signals collected during the study are selected for each gait cycle (EMG bins) and the RQA was conducted on each bin of data and the outcomes were combined by the use of SYNERGOS algorithm.

### 2.2. Protocol

The experiment was conducted during a single collection session at the Laboratory of Integrated Physiology located at the University of Houston. All subjects underwent an initial interview and assessment. The participants had not taken levodopa at least eight hours prior to participating in this study “off medication”. At the outset of the protocol, the participants were instructed to choose a comfortable walking speed they felt was sustainable for up to two minutes and closely approximated their walking pace during “community-based” activities [19]. The choice of this “comfortable” gait speed was identified over a range of speeds by changing the treadmill speed several times until the individual identified their unique self-selected speed. Participants then rested for five minutes. The initial walking speed was used as the starting treadmill speed during the “off” and “on” states of levodopa. The experiment started with the subject walking at the previous speed, which was then increased by 0.045 m/s every five strides. Participants were instructed to continue walking until they felt uncomfortable continuing or up to a maximum of 180 seconds. Participants then immediately took their Levodopa medication and rested for 45 minutes before repeating the protocol for the second trial, the “on-medication” condition.

### 2.3. Data Processing

EMG and kinematic data were analyzed using a customized Matlab script (Mathworks, USA R2010b). An initial surrogate testing was conducted according to methods presented elsewhere, to justify the use of RQA as a nonlinear tool in this experiment [18, 21–23]. Data processing via the SYNERGOS algorithm was conducted according to mathematical formulations described previously [18]. The calculated %DET of the EMG signal obtained from each muscle was used for computation of a single MMA value as generated by applying the SYNERGOS algorithm. The obtained single scalar value based on this processing technique indicates the overall activity among the set of multiple muscles and was used for subsequent statistical analysis.

### 2.4. Statistical Analysis

To analyze the efficiency of the SYNERGOS to detect the changes in MMA associated with levodopa intake (i.e., “off” or “on” state of levodopa) and changing gait speed, a restricted maximum likelihood linear mixed model was employed. The model included three fixed effects—speed, medication (“off” or “on”), and speed-by-medication interaction—and two random effects—subjects and measurement error (i.e., random within-subject variation). Although this analytical approach accounts for dependency, resulting from multiple measures within each subject similar to repeated-measures analysis of variance, it does not require equality in the number of measures for each subject. As each individual initiated the walking with their self-selected walking speed (different between the subjects) and finished the protocol when they felt uncomfortable continuing (different between subjects), the number of measures (i.e., gait speed increments) from each subject was different. The fixed effects were used to test the experiment hypotheses. The significance level was set at *p* ≤ 0.05, and analysis was conducted using SPSS 17.0.1 (SPSS Inc., Chicago, IL, USA).

## 3. Results

### 3.1. Surrogate Testing

Three surrogate testing algorithms were applied to each muscle within each gait cycle to justify the use of RQA in the EMG analysis for both conditions (i.e., off-medication and on-medication). These algorithms were based on (1) time shuffled surrogate testing (2) Fourier transform (FT), and (3) iterated amplitude adjusted Fourier transform surrogate testing (IAAFT). Results of the surrogate procedures for the condition in which the participants were off medication and after they were medicated were tested for potential differences. Under both conditions, the null hypothesis was rejected by reaching a discriminant statistic of more than 2 (*φ* > 2 and *p* < 5%). These results indicated that a more complex nonlinear pattern did indeed exist in EMG signals collected from the individuals, thereby justifying the use of RQA to investigate the changes in the signals.

**Figure 4:**
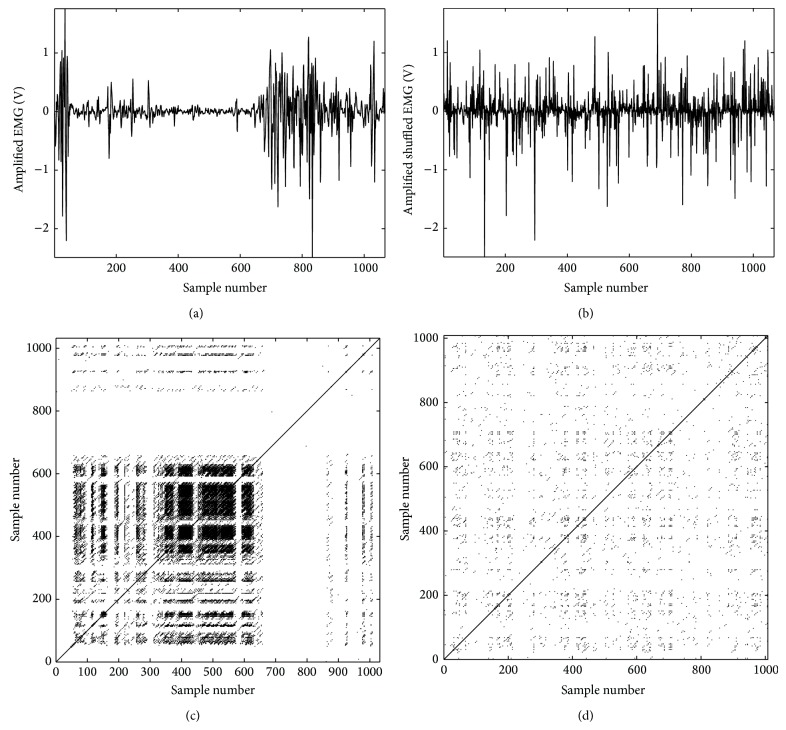
Neuromuscular activity of tibialis anterior (TA) muscle during a gait cycle depicted by EMG activity. (a) TA is active during the initial phase of gait cycle (heel contact), with a reduction of activity towards the plantar flexion position. From the midstance toward the terminal stance, the dorsiflexion of ankle is managed by higher activity of TA. The recurrence plots (RP) generated based on the original data are shown in graph (b) indicating the existence of a specific pattern in TA activity during the gait cycle. This pattern is depicted by several recurrent points located along particular diagonal lines which are parallel to the main diagonal line. The outcome of the RQA also verified the existence of the aforementioned pattern (%REC = 1.93, %DET = 34.80, radius = 1.92 (maximum scale), and ApEn = 0.63). (c) contains the randomized shuffled data of the signal shown in (a); (d) is the RP of the randomized signal which shows no significant determinism in the shuffled data. The time delayed dimensional data in RP are randomly scattered around the main diagonal line and the recurrent points are positioned along very short length. In addition, the outcome of RQA has shown significant reduction in determinism in the randomized data (%REC = 0.44, %DET *≅* 0, radius = 1.92 (maximum scale), and ApEn = 1.93). The significant drop in the determinism of the signal detected by decreasing %DET and increasing ApEn verified the nonlinear dynamics of the collected EMG signal.

The tibialis anterior acts as the primary ankle dorsiflexor. At heel contact, the ankle is positioned in a neutral or minimal plantar flexed angle (3 to 5 degrees). The plantar flexion ankle angle increases after heel contact, hence the reduction in TA activation. The neuromuscular activities of the TA increase from the midstance position throughout the terminal stance, resulting in ankle dorsiflexion. This pattern is shown in Figure 4(a) and the recurrence plot of the signal in which the deterministic patterns are shown is depicted in Figure 4(b). The shuffled EMG signal is shown in Figure 4(c) and the corresponding recurrent plot in Figure 4(d). The randomization of the signal resulted in scattered points within the recurrent plot; this did not demonstrate any specific pattern. The shuffling process destroyed the underlying pattern of the original signal. These outcomes indicate that indeed the use of higher order analysis such as RQA might be valuable to reveal nonlinear patterns embedded in the collected EMG signals.

### 3.2. SYNERGOS Analysis

The overall SYNERGOS index across all individuals and speeds whether on or off medication was 22.14 ± 9.42 (mean ± standard deviation). Data is summarized in Table 2. The assumption of normality was checked for the SYNERGOS indices calculated during off- and on-medication conditions using the Kolmogorov-Smirnov test. The SYNERGOS indices during both conditions demonstrated a normal distribution (off medication: *D*(151) = .047, *p* = 0.20, and on medication *D*(189) = 0.06, *p* = 0.097).

The medication intake significantly reduced the overall MMA quantified by SYNERGOS value, *F*(1, 255.11) = 13.834, *p* < 0.001, hence confirming the hypothesis of this experiment that the algorithm was able to detect subtle changes in the MMA due to levodopa intake.

**Figure 5:**
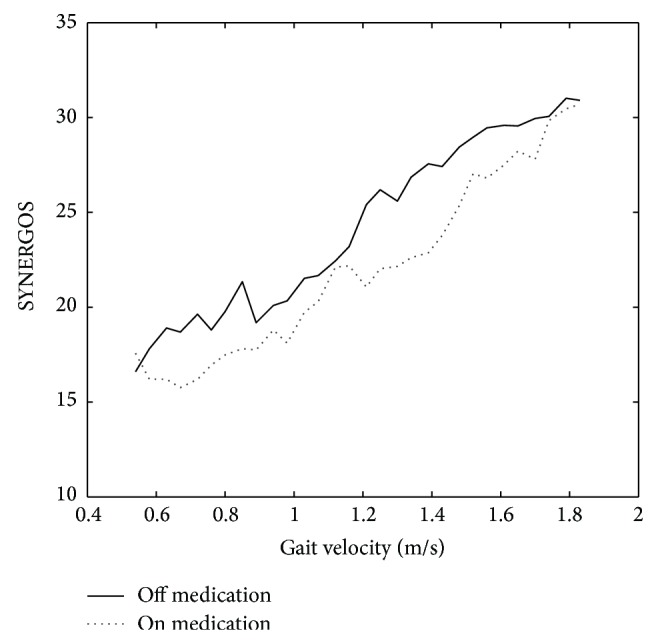
The mean values of the SYNERGOS indices were compared while on and off medication, during which lower MMA is associated with medication intake.

The outcome suggests that collective overall activity of the monitored muscles was decreased during on-medication condition when compared to the same subjects when performing the task while “off” medication. Additionally, the SYNERGOS index was able to reflect the subtle changes in MMA associated with a slight increase in walking speed (i.e., increase of 0.045 m/s, *F*(40, 255.091) = 13.834, *p* < 0.001), as shown in Figure 5.

The interaction effect of gait speed and medication intake was not significant, *F*(34, 255.017) = 48.075, *p* > 0.05, indicating the consistent behavior of the SYNERGOS index in detecting changes in the state of MMA during different pharmaceutical conditions.

## 4. Discussion

In this experiment, SYNERGOS was used to investigate the changes in the overall neuromuscular activation of individuals with Parkinson's disease in response to medication intake and walking speed. The premise of SYNERGOS is that it eventually can be used as a reliable and valid assessment algorithm in clinical settings to monitor the changes in MMA over time or as a screening tool to identify potential neuromuscular abnormalities early in the progression of a disease state.

The SYNERGOS algorithm detected a significant change in the quantified MMA after levodopa intake as indicated by lower SYNERGOS indices. The changes in the biomechanical aspects of the movement (i.e., more regularity of lower extremity [19]) suggested that these individuals employed different control strategies resulting in reduced MMA after medication intake. This may be due to the fact that less overall neuromuscular activities were recorded by EMG in each epoch (i.e., gait cycle) after medication intake. It is suggested that, in patients with Parkinson's disease, the reciprocal inhibition at the level of the spinal cord is compromised. During each movement, disynaptic Ia reciprocal inhibition function is to actively inhibit the antagonist motor neurons while reducing the inhibition of the agonists motor neurons. Parkinson's disease causes abnormalities in the function of disynaptic Ia reciprocal, hence resulting in the higher level of coactivation of agonist and antagonist muscles which ultimately thus increase the level of MMA in the Parkinson's disease individuals [24] which may lead to freezing gait and movement rigidity. Levodopa however is prescribed to overcome these symptoms by reducing the overall coactivity of the muscles, hence resulting in more optimal movement pattern. The current results suggest that Levodopa may in fact increase reciprocal inhibition and therefore decreases overall MMA.

During this experiment, participants performed a treadmill walking regimen in which the gait speed increased every five strides. The outcome indicated that the small changes in overall neuromuscular activities as a result of subtle change in the kinematics of the movement (i.e., gait speed) were reliably detected by the SYNERGOS algorithm. Previous studies have indicated that the stability of the human body decreases during higher gait speed which might be correlated with higher risk of fall and injury [25–27]. Muscular cocontraction is a strategy used to stiffen the joints resulting in the reduction of kinematic degrees of freedom to enhance stability during dynamic movements that may threaten postural stability [28]. During faster movements kinematics (velocity and accelerations) and kinetics (i.e., forces, torque, and momentum) parameters are altered at higher rates; therefore, a more reliable postural/movement strategy is required to ensure relatively quicker response to the variations imposed on the stability of the system. Thus, to compensate for the required energy and balance to provide more movement stability during faster gait, the CNS activates a higher number of motor units resulting in a higher level of MMA. Increasing SYNERGOS indices are compatible with the aforementioned observation of the motor control strategy.

There are some limitations to this study. Although SYNERGOS enables simplicity of EMG analysis for clinical measurement and monitoring, there is a trade-off with respect to some temporal aspects of muscular activation. To perform SYNERGOS analysis, epochs of signals (i.e., signals that are obtained from a specific cycle, such as gait cycle or squat cycle) are used; therefore, each SYNERGOS index represents an estimation of MMA during a specific epoch. This unit has less time resolution compared to the actual data. For example, if the EMG signals are collected at 1000 Hz, the data contains 1000 data points per second. If we assume an epoch with the length of one second, then only one SYNERGOS index representing all detectable muscle activation can be derived during that epoch. Although this process simplifies and reduces the amount of data to be monitored, it also limits the time resolution of the SYNERGOS index (e.g., 1000 samples versus 1 sample). However, this issue may provide the opportunity to record and store EMG signals for a much longer time without facing physical memory storage challenges. This will be valuable in longitudinal studies requiring data recording over a long period of time.

Additionally, in our experiment, participants were instructed to hold on to safety bars to further control their balance during the protocol. This might have added a systematic error to the results as the levodopa intake would change the overall neuromuscular activities in both the lower and the upper limbs. In this experiment, we did not measure the neuromuscular activities of the upper body; hence, it is impossible to draw a conclusion about the exact effect of the contribution of the upper body muscles to the changes in the neuromuscular activities of the lower body muscles while on and off medication. Although SYNERGOS was capable of detecting the significant decrease in MMA after levodopa intake, the results might have been altered systematically as the upper body control strategies were altered between the two conditions (off versus on medication). Finally, in our study we focused on the biomechanical aspects of the changes in the body in response to levodopa intake. The UPDRS score was assessed only after medication intake and we did not assess the score prior to levodopa intake. Future studies may be conducted aiming at gait characteristics and neuromuscular activation patterns when participants are secured via a harness not requiring additional handle bars.

## 5. Conclusion

In this experiment, we demonstrated significant changes in multiple muscle activation among individuals with Parkinson's disease in response to levodopa and changes in walking speed, assessed by SYNERGOS indices. The responsiveness, consistency, and simplicity of monitoring of SYNERGOS values indicate potential applicability of this algorithm in clinical settings to assess changes in the overall neuromuscular activity in individuals with Parkinson's disease as a result of therapeutic interventions.

## Figures and Tables

**Table 1:** Subjects' initial measurements.

Age (years)	Height (meters)	Mass (kg)	Walking speed (m/s)	UPDRS III score^*^	Hoehn and Yahr stage^**^
76 ± 6	1.7 ± 0.1	71.9 ± 12	0.62 ± 0.4	28.6 ± 4.6	2.8 ± 0.7

^*^Measured after the participants took medication.

^**^Measured prior to medication intake.

**Table 2:** The mean and standard deviation (SD) of the SYNERGOS index during off- and on-medication conditions.

	Average	SD
Off	23.74	9.17
On	20.86	9.44
